# Mapping and Identification of the Urine Proteome of Prostate Cancer Patients by 2D PAGE/MS

**DOI:** 10.1155/2014/594761

**Published:** 2014-08-20

**Authors:** Sanja Kiprijanovska, Sotir Stavridis, Oliver Stankov, Selim Komina, Gordana Petrusevska, Momir Polenakovic, Katarina Davalieva

**Affiliations:** ^1^Research Centre for Genetic Engineering and Biotechnology “Georgi D Efremov”, Macedonian Academy of Sciences and Arts, Krste Misirkov 2, 1000 Skopje, Macedonia; ^2^University Clinic for Urology, University Clinical Centre “Mother Theresa”, 1000 Skopje, Macedonia; ^3^Institute of Pathology, Medical Faculty, University of “St. Cyril and Methodius”, 1000 Skopje, Macedonia

## Abstract

Proteome analysis of the urine has shown that urine contains disease-specific information for a variety of urogenital system disorders, including prostate cancer (PCa). The aim of this study was to determine the protein components of urine from PCa patients. Urine from 8 patients with clinically and histologically confirmed PCa was analyzed by conventional 2D PAGE. The MS identification of the most prominent 125 spots from the urine map revealed 45 distinct proteins. According to Gene Ontology, the identified proteins are involved in a variety of biological processes, majority of them are secreted (71%), and half of them are enzymes or transporters. Comparison with the normal urine proteome revealed 11 proteins distinctive for PCa. Using Ingenuity Pathways Analysis, we have found 3 proteins (E3 ubiquitin-protein ligase rififylin, tumor protein D52, and thymidine phosphorylase) associated with cellular growth and proliferation (*p* = 8.35 × 10^−4^ − 3.41 × 10^−2^). The top network of functional associations between 11 proteins was *Cell Death and Survival, Cell-To-Cell Signaling and Interaction, and System Development and Function *(*p* = 10^−30^). In summary, we have created an initial proteomic map of PCa patient's urine. The results from this study provide some leads to understand the molecular bases of prostate cancer.

## 1. Introduction

Urine has become one of the most attractive biofluids in clinical proteomics because it can be obtained in large quantities, can be sampled noninvasively, and does not undergo significant proteolytic degradation compared with other biofluids [1]. The urine contains water, glucose, salt, and proteins derived from plasma or the urogenital tract. It can be viewed as modified ultrafiltrate of plasma combined with proteins derived from kidney and urinary tract, with protein concentration approximately 1000-fold lower than in plasma itself [2].

Even though the urinary proteome is much less complex than the plasma proteome, it contains high number of proteins. The urinary proteome has been studied by almost any proteomics technology. The first proteomic profiling of the normal urine was performed in 1979 using two-dimensional electrophoresis (2D) [3]. Afterwards, 2D, liquid chromatography (LC) and capillary electrophoresis (CE), all of them coupled to mass spectrometry (MS), have been used extensively in the proteomics definition of the urine. With the advent of the high throughput proteomics platforms consisting of 1D SDS-PAGE or LC coupled with high resolution mass spectrometers such as LTQ-FT and LTQ-Orbitrap, the number of detected proteins in healthy urine reached from 1310 to 1823, depending on the technology used [4–6]. The number of detected proteins in normal urine using 2D PAGE/MS is relatively lower compared to the proteomics platforms mentioned above [7]. This is mainly due to two reasons: lack of ability to cope with the broad dynamic range of complex samples and with hydrophobic proteins. However, 2D PAGE/MS is still an indispensable platform in proteomics, particularly for the assessment of the molecular mass of any protein or protein fragments and posttranslational modifications [8].

Qualitative and quantitative changes in urinary proteome often point out to disease-related changes starting from urogenital diseases but also to some systemic diseases [9]. Proteomic analysis of urine has shown that it contains disease-specific information for various diseases. Up till now, urine has been used as a source of biomarkers for a number of kidney diseases and cancers related to the urogenital system such as bladder and prostate cancer, as well as various nonnephrological/urogenital diseases such as preeclampsia, stroke, coronary artery diseases, heart failure, acute appendicitis in children, and graft-versus-host disease reviewed extensively elsewhere [9, 10].

In this study, we describe proteomic map of urine from prostate cancer (PCa) patients using 2D PAGE/MS profiling. The determination of urinary proteome of PCa patients has created an initial database which can be used for comparison to normal urinary proteome database as well as to various cancer diseases urine proteome databases. The results from this study broaden the current knowledge in the field of urinary proteomics and provide some leads to understand the molecular bases of prostate cancer pathophysiology.

## 2. Materials and Methods

### 2.1. Urine Samples

We analyzed 8 urine samples from patients with PCa prostate obtained from the University Clinic for Urology, University Clinical Centre “Mother Theresa,” Skopje, Republic of Macedonia. Informed consent for the use of these samples for research purposes was obtained from the patients in accordance with the Declaration of Helsinki. The study has been approved by the Ethics Committee of the Macedonian Academy of Sciences and Arts.

Patient's clinical records including histology grading, tumor stage, and preoperative prostate-specific antigen (PSA) serum levels were reviewed to preselect the urine samples. Eight urine samples from patients with clinically confirmed and histologically graded tumors were chosen from the urine archive (Table 1), for the 2D PAGE/MS analysis. The preoperative serum PSA levels ranged from 4.6 to 50.0 ng/mL (mean PSA = 17.1 ± 16.6). The mean age of the selected patients was 69 ± 6.3 years and the mean Gleason score was 7.0 ± 1.1.

The first morning urine (3–10) mL was collected from the patients prior to clinical intervention and stored on ice for short period (<1 h). Samples were centrifuged at 1000 ×g, for 10 min, to remove cell debris and casts, aliquoted in 1.5 mL tubes, and stored at −80°C until use.

### 2.2. Preparation of Total Protein Extract from Urine

The stored urine samples were thawed and, for each sample, proteins were isolated in triplicate from 100 *μ*L urine using 2D Clean-UP Kit (GE Healthcare) according to the manufacturer's instructions. The pellets from each replicate were dissolved in 10 *μ*L of UTC buffer (8 M urea, 2 M thiourea, 4% CHAPS), pooled together for each sample, quantified by Bradford method [11] in duplicate against a standard curve of bovine serum albumin (BSA), and stored at −80°C until use.

### 2.3. 2D PAGE

Pooled samples of total protein extract from urine were used. Equal amounts of proteins from each of the 8 samples were pooled to the total of 400 *μ*g of protein per gel. Pooled samples were analyzed in three technical replicates by conventional 2D PAGE analysis on immobilized pH gradient strips Immobiline DryStrip pH 4–7 (GE Healthcare, Little Chalfont, Buckinghamshire, UK) and 12.5% SDS PAGE subsequently. For the immobilized pH gradient strips pH 4–7, we have used the rehydration buffer consisting of 8 M urea, 2 M thiourea, 2% (w/v) CHAPS, 10 mM DTT, 1.2% (v/v) IPG-buffer pH 4–7, and trace of bromophenol blue. Isoelectric focusing of the rehydrated 24 cm immobilized pH gradient strips was performed on Ettan IPGphor 3 system (GE Healthcare, Little Chalfont, Buckinghamshire, UK). The strips were focused until total of 64.5 kVh was reached. The focused proteins in the immobilized pH gradient strips were immediately equilibrated in two incubation steps, each lasting 15 min, at room temperature. In the first step, the equilibration buffer (6 M urea, 2% (w/v) SDS, 30% (v/v) glycerol, 50 mM tris, pH 8,6) was supplemented with 1% (w/v) DTT for reduction, followed by alkylation in the same buffer containing 4.7% (w/v) iodoacetamide instead of DTT. The second dimension was carried using Ettan DALTsix system (GE Healthcare, Little Chalfont, Buckinghamshire, UK) at 2.5 W per gel for 30 min, followed by 16 W/gel for 4 h.

### 2.4. 2D PAGE Imaging and Analysis

The gels were stained with Coomassie G-250. Gels were fixed in 30% (v/v) ethanol and 2% (v/v) phosphoric acid for 30 min with two exchanges of the fixing solution, washed three times with 2% (v/v) phosphoric acid for 10 min each, balanced in prestaining buffer (12% (w/v) (NH_4_)_2_SO_4_, 2% (v/v) phosphoric acid, and 18% (v/v) ethanol) for another 30 min, and stained in staining solution (0.01% (w/v) CBB G-250, 12% (w/v) (NH_4_)_2_SO_4_, 2% (v/v) phosphoric acid, and 18% (v/v) ethanol) for 72 h. The gels were stored in the staining solution until the spots of interests were manually picked.

The gels were scanned on an Ettan DIGE imager (GE Healthcare) and the resulting images were analyzed with ImageMaster 2D Platinum 7.0 (GE Healthcare, Little Chalfont, Buckinghamshire, UK) software. ImageMaster Platinum values of smooth, minimum area and saliency were 2, 5, and 50, respectively. Exclusion filter (vol >450) was applied to remove artificial spots and dust particles.

### 2.5. Mass Spectrometry: Ingel Tryptic Digestion

Ingel digestion was carried out manually with trypsin. Spots were first destained two times with a mixture of 50% (v/v) ACN for 15 min each and then once with 100 mM NH_4_HCO_3_ and 50% (v/v) ACN for 15 min. Spots were dried in vacuum centrifuge and then reduced with 100 mM NH_4_HCO_3_ containing 10 mM DTT for 45 min at 56°C and then alkylated with 54 mM iodoacetamide in 100 mM NH_4_HCO_3_ for 30 min in the dark, at room temperature. Gels pieces were washed with 100 mM NH_4_HCO_3_, shrunk with 50% ACN for 15 min, and dried in vacuum centrifuge. Gel particles were rehydrated with 20 *μ*L of 0.01 *μ*g/*μ*L trypsin proteomics grade (Roche Diagnostics GmbH) in digestion buffer (95% 50 mM NH_4_HCO_3_/5% ACN) for 45 min at room temperature. The remaining enzyme supernatant was replaced with one gel volume of the digestion buffer and digestion was carried out at 37°C, overnight. After digestion, peptides were collected in separate tube, extracted once with 20 *μ*L of 50% ACN and twice with a mixture of 50% ACN/5% formic acid, dried in vacuum centrifuge, and reconstituted in 10 *μ*L of 0.1% TFA.

### 2.6. Mass Spectrometry: Identification

For MS analysis, peptides were purified using ZipTip_C18_ (Millipore Corporation) following the manufacturer's instructions and eluted in 2-3 *μ*L of CHCA (4 mg/mL in 50% ACN/0.1% TFA) directly onto a MALDI target plate (Shimadzu Biotech Kratos Analytical). Droplets were allowed to dry at room temperature. Samples analysis was performed using AXIMA Performance MALDI-TOF-TOF mass spectrometer (Shimadzu Biotech Kratos Analytical). Spectra acquisition and processing were performed using the MALDI-MS software (Shimadzu Biotech Kratos Analytical) version 2.9.3.20110624 in positive reflectron mode at mass range 1–5000 Da with a low mass gate at 500 Da and pulsed extraction optimized at 2300 Da. External calibration was performed based on monoisotopic values of five well-defined peptides: bradykinin fragment 1–5, angiotensin II human, [Glu1]-gibrinopeptide B human, adrenocorticotropic hormone fragment 1–17 human, and adrenocorticotropic hormone fragment 7–38 human (Sigma-Aldrich). External calibration mix (500 fmol/*μ*L) was diluted with the matrix in ratio 1 : 1 and applied onto the MALDI target plate at final concentration of 250 fmol per spot. Each mass spectrum was acquired by 500 laser profiles (five pulses per profile) collected across the whole sample. After filtering tryptic-, keratin-, and matrix-contaminant peaks, the resulting monoisotopic list of m/z values was submitted to the search engine Mascot (version 2.4.01, MatrixScience, UK) searching all human proteins and sequence information from Swiss-Prot (version 2014_05, 20265 sequences) and NCBInr (version 20140323, 276505 sequences). The following search parameters were applied: fixed modification-carbamidomethylation and variable modifications-methionine oxidation and N-terminal acetylation. Up to 1 missed tryptic cleavage was permitted and peptide mass tolerance of ±0.40 Da was used for all mass searches. Positive identification was based on a Mascot score greater than 56, above the significance level (*P* < 0.05). The reported proteins were always those with the highest number of peptide matches.

### 2.7. Functional Characterization of the Identified Proteins

For an overview of the cellular localization, molecular function, and biological processes in which identified proteins are included, we used the UniProt Knowledgebase (UniProtKB) and Gene Ontology (GO) database. The accession numbers of the identified proteins were imported into Ingenuity Pathway Analysis (IPA) (Ingenuity Systems, USA) and functionally assigned to canonical pathways and the most significant networks generated from previous publications and public protein interaction databases. A* P* value calculated with the right-tailed Fisher's exact test was used to yield a network's score and to rank networks according to their degree of association with our data set.

## 3. Results

For this study, we selected urine samples from 8 patients with clinically and histologically confirmed PCa cancer (Table 1). The mean age of the selected patients was 69 years (±6.3), the mean Gleason score was 7.0 (±1.1), and the mean preoperative serum PSA level was 17.1 (±16.6). The average amount of purified proteins from 100 *μ*L urine ranged from 18.2 to 29.8 *μ*g. The analysis of each patient urine proteome required substantial number of isolations and 2D PAGE gels for analysis. Therefore, we used pooled samples of total protein extract from 8 patients. The pooled samples also give more relevant picture of the PCa patient's urine proteome.

**Figure 1:**
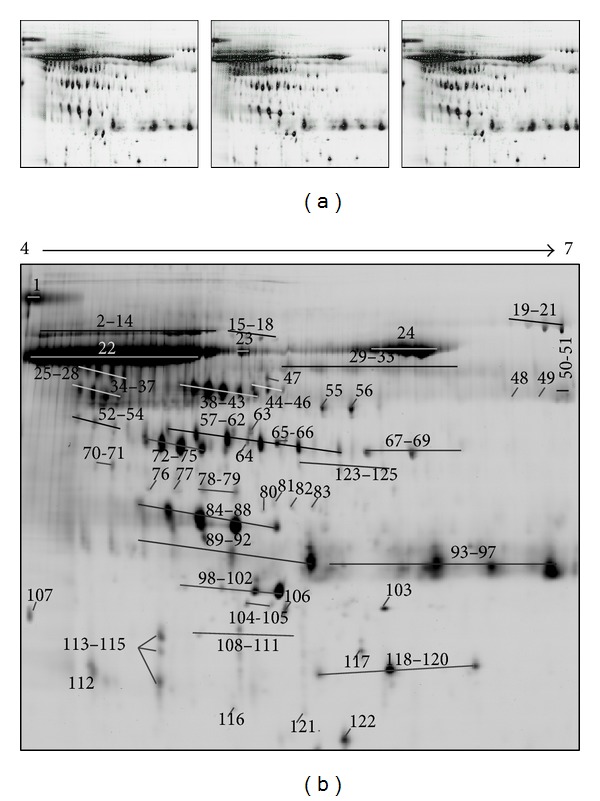
2D maps of the urine proteome from PCa patients obtained by 2D electrophoresis using IEF on pH 4–7 IPG strip and 12.5% SDS-PAGE. (a) Images of the three technical replicates of the pooled urine protein samples. The detected spots in the images are represented with green dots. Overall, 948 spots were reproducibly visualised in the three maps. (b) Representative 2D map of the urine proteome. All identified protein spots are marked with numbered arrows. Details of these proteins identified by MALDI MS are tabulated in Table 2.

Following 2D PAGE and staining, 1085 ± 110 spots were detected and 948 of them were reproducibly visualized in the three technical replicates (Figure 1(a)). The most prominent 125 spots were picked up for the MS analysis. The position of these spots in the 2D urine map is shown in Figure 1(b). The MS identification of these spots revealed that they belong to 45 distinct proteins (Table 2). A number of proteins were presented on 2D map as a horizontal row of multiple spots (with small changes in pI and molecular weight), likely caused by variable posttranslational modifications (PTMs). All identified proteins were in the expected molecular size (14–80 kDa) and pI value (4.93 to 8.07) and the majority of it were high-molecular weight proteins (>30 kDa). Representative 2D map showed no evidence of protein degradation that usually presents as vertical streaking of the protein stain.

**Figure 2:**
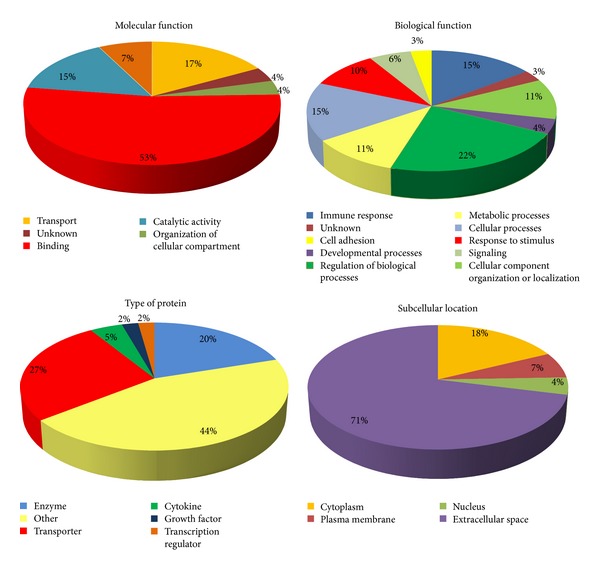
Classification of the identified proteins in urine of PCa patients. The molecular function, biological processes in which they are involved, subcellular location, and type of protein were assessed by Gene Ontology search.

The identified proteins from PCa patient's urine were classified by subcellular location, molecular function, biological function, and type of protein using the available data from the UniProt Knowledgebase (UniProtKB) and Gene Ontology (GO) database (Figure 2). Regarding molecular function, 53% of all identified proteins in PCa patient's urine have binding function, followed by transport and catalytic activity. Approximately half of the identified proteins are involved in regulation of biological processes, developmental processes, and cellular processes. Half of the proteins were enzymes and transporters and the rest belonged to transcription regulators, growth factors, cytokines, and other protein types. The majority of the identified proteins (71%) were secreted and had extracellular space location.

Comparison of the identified 45 proteins with proteins identified in normal urine by 2D PAGE/MS [7] showed that 34 proteins are found in the normal urine as well, while 11 have not been reported. The functional characteristics of the 11 proteins are given in Table 3.

**Figure 3:**
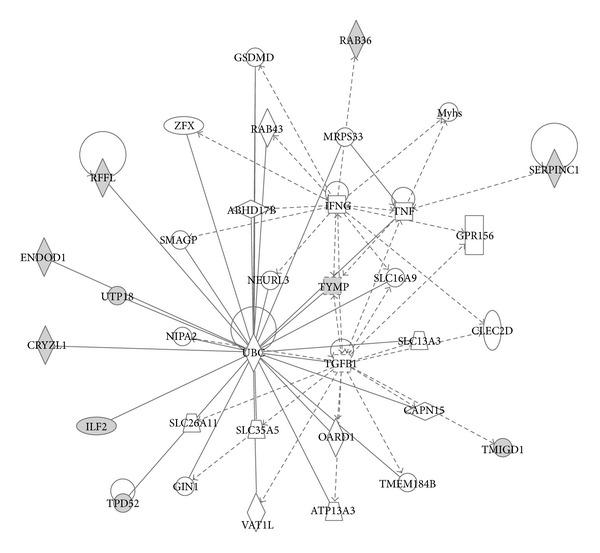
Network associated with the 11 urinary proteins from patients with PCa according to IPA. Top protein network of functional associations between proteins was Cell Death and Survival, Cell-To-Cell Signaling and Interaction, and System Development and Function with score 30 (*p* = 10^−30^). The proteins are connected through four major nodes: ubiquitin (UBC) and cytokines, TNF, TGFB1, and IFNG. The proteins identified in this study are represented with gene names (for protein name, please see Table 3) and coloured in gray. The network is graphically displayed with proteins as nodes and the biological relationships between the nodes as lines. Different shapes represent the functional classes of proteins. The length of a line reflects published evidence supporting the node-to-node relationship concerned.

Using Ingenuity Pathways Analysis (IPA) classification and networking, we found out that some of the 11 identified proteins in PCa are significantly associated with cancer and organism injury and abnormalities diseases and disorders. Four proteins (antithrombin-III, transmembrane and immunoglobulin domain-containing protein 1, tumor protein D52, and thymidine phosphorylase) are associated with different types of cancers (*p* = 4.17 × 10^−4^ − 3.21 × 10^−2^), while 2 proteins (antithrombin-III and thymidine phosphorylase) are associated with organism injury and abnormalities (*p* = 8.35 × 10^−4^ − 4.54 × 10^−2^). In the molecular and cellular function classification, we have found 3 proteins (E3 ubiquitin-protein ligase rififylin, tumor protein D52, and thymidine phosphorylase) associated with cellular growth and proliferation (*p* = 8.35 × 10^−4^ − 3.41 × 10^−2^). The top protein network of functional associations between proteins was Cell Death and Survival, Cell-To-Cell Signaling and Interaction, and System Development and Function with score 30 (*p* = 10^−30^). The network encompassed 10 from 11 proteins closely connected through four major nodes: ubiquitin C (UBC), tumor necrosis factor (TNF), transforming growth factor beta 1 (TGFB1), and interferon gamma (IFNG) (Figure 3).

## 4. Discussion

Determination of protein map and composition of PCa patient's urine may lead to an increased understanding of cancer pathophysiology. Using 2D PAGE/MALDI-TOF, we have identified a total of 125 protein spots belonging to 45 unique proteins in PCa patient's urine. According to the molecular and functional data for these proteins, they can be classified into several groups:* kidney secretory and structural proteins*: uromodulin (formerly Tamm-Horsfall protein), vesicular integral-membrane protein (VIP), gelsolin, and actin cytoplasmic 1 and 2, basement membrane-specific heparin sulphate proteoglycan core protein;* serum constitutional and transport proteins*: albumin, vitamin D binding protein, leucine-rich *α*2 glycoprotein, transthyretin, plasma retinol-binding protein, haptoglobin *Β* chain, ubiquitin, *α*-1 acid glycoprotein 1, and tumor protein D52;* coagulation factors*: fibrinogen *α* chain, kininogen-1, fibrinogen *β* chain, fibrinogen *γ* chain, CD59 glycoprotein, and antithrombin-III;* complement fraction*s: mannan-binding lectin serine protease, immunoglobulins with heavy and light chains, secreted and transmembrane protein 1, protein AMBP, and transmembrane and immunoglobulin domain-containing protein 1;* proteases and inhibitors*: *α*1 antitrypsin and *α*1 antitrypsin fragment, *α*1 antichymotrypsin, and inter-*α* trypsin inhibitor heavy chain H4;* enzymes*: prostaglandin-H2-isomerase, alpha-amylase 1, thymidine phosphorylase, endonuclease domain-containing 1 protein, quinone oxidoreductase-like protein 1, E3 ubiquitin-protein ligase rififylin, and Ras-related protein Rab-36;* metal binding proteins*: serum transferrin, *α*-HS-glycoprotein, and zinc-*α*-2-glycoprotein;* transcriptional regulators*: U3 small nucleolar RNA-associated protein 18 and interleukin enhancer-binding factor 2; and* lipoprotein metabolism*: apolipoprotein.

Regarding the subcellular location of the identified proteins, our analysis revealed that extracellular proteins and plasma membrane proteins represent the majority in the PCa patient's urine. This was expected for two reasons: first, the urine is in direct contact with several glands in the male urinary tract, and, second, substantial fraction of the urinary proteins is derived from plasma [4, 7]. Among diverse biological functions in which the identified set of urinary proteins is involved, signal transducer activity was unexpected as this function was not present in the observed proportion in proteins from the plasma proteome [4]. On the other hand, it was expected that the PCa patient's urine contains substantial amount of immune response proteins, proteins involved in response to stimuli, signalling and adhesion molecules, as cancers may be considered a state of constant inflammation of the organism [12].

Thirty-four proteins in our study have been reported as constituents of the normal urine, while 11 proteins (antithrombin-III, alpha-amylase 1, U3 small nucleolar RNA-associated protein 18 homolog, thymidine phosphorylase, endonuclease domain-containing 1 protein, E3 ubiquitin-protein ligase rififylin, quinone oxidoreductase-like protein 1, interleukin enhancer-binding factor 2, transmembrane and immunoglobulin domain-containing protein 1, Ras-related protein Rab-36, and Tumor protein D52) were not reported in the normal urine proteome [7]. Of them, thymidine phosphorylase, E3 ubiquitin-protein ligase, and tumor protein D52 are involved in processes of angiogenesis, tumor growth, or metastasis and the rest are proteins with different physiological functions and no reported involvement in cancer development or progression. IPA analysis of the 11 proteins also pointed out these 3 proteins as significantly associated with cellular growth and proliferation.

Thymidine phosphorylase (TYMP) is an enzyme involved in pyrimidine metabolism and also known to be a platelet-derived endothelial cell growth factor (PD-ECGF). TYMP is overexpressed in various tumors including prostate cancer and plays an important role in angiogenesis, tumor growth invasion, and metastasis [13]. E3 ubiquitin-protein ligase (RFFL) has been implicated in regulation of p53 tumor suppressor stability [14, 15]. Major physiological function of RFFL is promotion of p53 destruction and, as a result of it, it is frequently found overexpressed in a variety of human cancers [16]. The tumor protein D52 (TPD52) is frequently and strongly upregulated in many human cancer types and this trend is observed in various urogenital cancers among which is prostate cancer as well [17].

Furthermore, IPA analysis pointed out functional associations between the 11 proteins. The network encompassed 10 out of 11 proteins connected through four nodes: ubiquitin C (UBC), tumor necrosis factor (TNF), transforming growth factor beta 1 (TGFB1), and interferon gamma (IFNG). Ubiquitination has been associated with protein degradation, DNA repair, cell cycle regulation, kinase modification, endocytosis, and regulation of other cell signaling pathways [18, 19]. Human neurodegenerative, infectious diseases and tumorigenesis have been associated with alterations in ubiquitin pathways [20]. Ubiquitin has been detected in a variety of normal and cancerous tissues. Variations in the distribution and intensity of ubiquitin in benign and malignant conditions of the human prostate have been observed, leading to conclusion that it has implication in tumor pathogenesis of prostate cancer [21]. Our results are in concordance with this finding since seven out of ten proteins in the network, found in PCa, are directly connected to UBC.

The rest of the regulatory nodes in the network are represented by cytokines (TFN, TGFB1, and IFNG). Cytokines are regulators of host responses to infection, immune responses, inflammation, and trauma [22]. The biological activity of these cytokines is conventionally associated with antitumor mechanisms during cell-mediated adaptive immune response. Despite this, a number of reports suggest that their role in carcinogenesis is complex, having both tumor suppressor and oncogenic activities [23–25].

The interaction of ubiquitin, cytokines, and urine proteins found in PCa patients in this study, as proposed by IPA network, having in mind dual nature of cytokines and ubiquitin in the cancer progression, may lead to deeper understanding of prostate cancer pathogenesis. The possible role of these proteins and their connection with the signal transduction cascade of prostate cancer remains to be solved in the future.

## 5. Conclusions

In summary, we have created an initial proteomic map of PCa patient's human urine. The most prominent spots were successfully identified and analyzed in context of prostate cancer. Comparison with other published studies analyzing normal urine proteome pointed out several proteins that might have some role in the pathogenesis of prostate cancer. Moreover, IPA analysis showed significant association of our proteins with cancer and cellular growth and proliferation. The attempts to identify more low-abundant proteins in the urine from PCa patients by different strategies as well as comparison with urinary proteome from different cancer are underway. Although the presented urinary proteome map from patients with PCa showed limited number of proteins, the information regarding their position, molecular mass, possible posttranslational modifications, and presence of different protein fragments are useful addition to the present knowledge and provide some leads to understand the molecular bases of prostate cancer pathophysiology.

## Figures and Tables

**Table 1:** Clinical information of patients used to generate urine samples included in the study together with their PSA levels, histology grading and tumor stage.

Sample number	Patient number	Age	Diagnosis	Tumor stage	Gleason score	Preoperative PSA (ng/mL)
1	PC-22	74	PCA	pT2c pN0 pM0	7 (3 + 4)	8.3
2	PC-24	72	PCA	pT2c pN0 pM0	7 (3 + 4)	5.5
3	PC-27	78	PCA	pT2c pN0 pM0	5 (2 + 3)	4.6
4	PC-28	60	PCA	pT2c pN0 pM0	7 (3 + 4)	5.2
5	PC-31	65	PCA	pT3b pN1 pM0	7 (3 + 4)	8.3
6	PC-35	74	PCA	pT2c pN0 pM0	7 (3 + 4)	22.6
7	PC-39	64	PCA	pT2b pN1 pM0	9 (4 + 5)	50.0
8	PC-48	65	PCA	pT2c pN0 pM0	7 (3 + 4)	32.2

**Table 2:** List of the all protein spots from the PCa urinary proteome identified by MS.

Spot number	Protein name	SwissProt accession number	Mw (kDa)	pI	Mascot protein score	*P* value	Number of the matched peptides	% of sequence coverage
1	Uromodulin OS = *Homo sapiens* GN = UMOD PE = 1 SV = 1	UROM_HUMAN	72.64	5.05	158	3.2*E* − 12	18	22
2–14, 19–21	Serotransferrin OS = *Homo sapiens* GN = TF PE = 1 SV = 3	TRFE_HUMAN	79.29	6.8	243	1.0*E* − 20	22	40
15–18	Alpha-1B-glycoprotein OS = *Homo sapiens* GN = A1BG PE = 1 SV = 4	A1BG_HUMAN	54.79	5.56	93	9.3*E* − 06	12	35
22–24	Serum albumin OS = *Homo sapiens* GN = ALB PE = 1 SV = 2	ALBU_HUMAN	71.31	5.92	246	5.1*E* − 21	26	48
25–28	Alpha-1-antichymotrypsin OS = *Homo sapiens* GN = SERPINA3 PE = 1 SV = 2	AACT_HUMAN	47.79	5.33	82	3.4*E* − 05	12	31
	Kininogen-1 OS = *Homo sapiens* GN = KNG1 PE = 1 SV = 2	KNG1_HUMAN	72.99	6.34	82	3.6*E* − 05	13	19
29–33	Ig alpha-1 chain C region OS = *Homo sapiens* GN = IGHA1 PE = 1 SV = 2	IGHA1_HUMAN	38.49	6.08	62	1.3*E* − 02	5	18
34–37	Alpha-2-HS-glycoprotein OS = *Homo sapiens* GN = AHSG PE = 1 SV = 1	FETUA_HUMAN	40.00	5.43	95	6.0*E* − 06	15	43
38–43	Alpha-1-antitrypsin OS = *Homo sapiens* GN = SERPINA1 PE = 1 SV = 3	A1AT_HUMAN	46.89	5.37	194	8.1*E* − 16	23	53
44–46	Vitamin D-binding protein OS = *Homo sapiens* GN = GC PE = 1 SV = 1	VTDB_HUMAN	54.52	5.40	135	6.4*E* − 10	12	50
47	Antithrombin-III OS = *Homo sapiens* GN = SERPINC1 PE = 1 SV = 1	ANT3_HUMAN	53.02	6.32	85	6.1*E* − 05	8	21
48	Alpha-amylase 1 OS = *Homo sapiens* GN = AMY1A PE = 1 SV = 2	AMY1_HUMAN	58.41	6.47	82	1.2*E* − 03	6	15
49, 67–69	Fibrinogen beta chain OS = *Homo sapiens* GN = FGB PE = 1 SV = 2	FIBB_HUMAN	56.57	8.54	62	1.3*E* − 03	8	19
50-51	U3 small nucleolar RNA-associated protein 18 homolog OS = *Homo sapiens* GN = UTP18 PE = 1 SV = 3	UTP18_HUMAN	62.42	8.93	75	6.6*E* − 04	6	13
52–54	Leucine-rich alpha-2-glycoprotein OS = *Homo sapiens* GN = LRG1 PE = 1 SV = 2	A2GL_HUMAN	38.38	6.45	66	5.3*E* − 03	4	18
55	Fibrinogen gamma chain OS = *Homo sapiens* GN = FGG PE = 1 SV = 3	FIBG_HUMAN	52.10	5.37	164	8.1*E* − 13	17	47
56	Thymidine phosphorylase OS = *Homo sapiens* GN = TYMP PE = 1 SV = 2	TYPH_HUMAN	50.32	5.36	64	7.4*E* − 03	5	25
57–62, 118–120	Haptoglobin OS = *Homo sapiens* GN = HP PE = 1 SV = 1	HPT_HUMAN	45.86	6.13	126	5.1*E* − 09	12	27
63	Gelsolin OS = *Homo sapiens* GN = GSN PE = 1 SV = 1 (fragment)	GELS_HUMAN	86.04 (52.48)	5.90 (5.34)	80	2.0*E* − 03	7	16
64	Apolipoprotein A-IV OS = *Homo sapiens* GN = APOA4 PE = 1 SV = 3	APOA4_HUMAN	45.37	5.28	82	1.2*E* − 04	8	22
65-66	Actin, cytoplasmic 1 OS = *Homo sapiens* GN = ACTB PE = 1 SV = 1	ACTB_HUMAN	42.05	5.29	64	7.0*E* − 03	7	24
	Actin, cytoplasmic 2 OS = *Homo sapiens* GN = ACTG1 PE = 1 SV = 1	ACTG_HUMAN	42.05	5.29	64	7.0*E* − 03	7	24
70-71	Fibrinogen alpha chain OS = *Homo sapiens* GN = FGA PE = 1 SV = 2 (fragment)	FIBA_HUMAN	95.65 (50)	5.7 (4.65)	91	1.6*E* − 05	10	15
72–75	Zinc-alpha-2-glycoprotein OS = *Homo sapiens* GN = AZGP1 PE = 1 SV = 2	ZA2G_HUMAN	34.46	5.71	104	8.1*E* − 07	8	29
76	Endonuclease domain-containing 1 protein OS = *Homo sapiens* GN = ENDOD1 PE = 1 SV = 2	ENDD1_HUMAN	55.72	5.55	104	8.1*E* − 07	9	20
77	E3 ubiquitin-protein ligase rififylin OS = *Homo sapiens* GN = RFFL PE = 1 SV = 1	RFFL_HUMAN	41.74	5.33	59	2.7*E* − 02	5	26
78-79	Inter-alpha-trypsin inhibitor heavy chain H4 OS = *Homo sapiens* GN = ITIH4 PE = 1 SV = 4 (fragment)	ITIH4_HUMAN	103.52 (45)	6.51 (5.15)	88	2.9*E* − 05	11	13
80	Quinone oxidoreductase-like protein 1 OS = *Homo sapiens* GN = CRYZL1 PE = 1 SV = 2	QORL1_HUMAN	39.07	5.49	66	4.9*E* − 02	4	12
81	Interleukin enhancer-binding factor 2 OS = *Homo sapiens* GN = ILF2 PE = 1 SV = 2	ILF2_HUMAN	43.26	5.19	62	3.0*E* − 02	5	19
82	Vesicular integral-membrane protein VIP36 OS = *Homo sapiens* GN = LMAN2 PE = 1 SV = 1	LMAN2_HUMAN	40.54	6.46	80	1.9*E* − 04	7	21
83	Transmembrane and immunoglobulin domain-containing protein 1 OS = *Homo sapiens* GN = TMIGD1 PE = 2 SV = 1	TMIG1_HUMAN	29.62	8.07	62	2.0*E* − 02	6	20
84–88	Protein AMBP OS = *Homo sapiens* GN = AMBP PE = 1 SV = 1	AMBP_HUMAN	39.87	5.95	130	2.0*E* − 09	15	37
89–92	Prostaglandin-H2 D-isomerase OS = *Homo sapiens* GN = PTGDS PE = 1 SV = 1	PTGDS_HUMAN	21.24	7.66	64	7.7*E* − 03	8	33
93–97	Ig kappa chain C region OS = *Homo sapiens* GN = IGKC PE = 1 SV = 1 (immunoglobulin kappa light chain VLJ region)	IGKC_HUMAN	28.82	6.73	77	5.8*E* − 03	7	29
98–102	Apolipoprotein A-I OS = *Homo sapiens* GN = APOA1 PE = 1 SV = 1	APOA1_HUMAN	30.75	5.56	116	5.1*E* − 08	16	46
103	Basement membrane-specific heparan sulphate proteoglycan core protein (perlecan) chain A, laminin-G-like domain 3 from human perlecan	PGBM_HUMAN	468.83 (20.65)	6.06 (5.47)	204	1.1*E* − 15	15	88
104-105	Ras-related protein Rab-36 OS = *Homo sapiens* GN = RAB36 PE = 2 SV = 2	RAB36_HUMAN	36.81	8.05	63	9.7*E* − 03	4	23
106	Tumor protein D52 OS = *Homo sapiens* GN = TPD52 PE = 1 SV = 2	TPD52_HUMAN	24.35	4.79	63	1.1*E* − 02	4	22
107	Alpha-1-acid glycoprotein 1 OS = *Homo sapiens* GN = ORM1 PE = 1 SV = 1	A1AG1_HUMAN	23.72	4.93	64	8.8*E* − 03	4	21
108–111	Retinol-binding protein 4 OS = *Homo sapiens* GN = RBP4 PE = 1 SV = 3	RET4_HUMAN	23.34	5.76	109	2.6*E* − 07	9	49
112–115	CD59 glycoprotein OS = *Homo sapiens* GN = CD59 PE = 1 SV = 1	CD59_HUMAN	14.79	6.02	61	1.6*E* − 02	5	28
116	Mannan-binding lectin serine protease 2 OS = *Homo sapiens* GN = MASP2 PE = 1 SV = 4 (chainA, human MBL-associated protein 19)	MASP2_HUMAN	77.19 (19.53)	5.44 (5.75)	127	5.5*E* − 08	8	37
117.121	Secreted and transmembrane protein 1 OS = *Homo sapiens* GN = SECTM1 PE = 1 SV = 2	SCTM1_HUMAN	27.30	7.00	75	6.1*E* − 04	6	22
122	Transthyretin OS = *Homo sapiens* GN = TTR PE = 1 SV = 1	TTHY_HUMAN	15.99	5.52	160	2.0*E* − 12	10	73
123–125	Uncharacterized protein KIAA2012 OS = *Homo sapiens* GN = KIAA2012 PE = 2 SV = 1 (fragment)	K2012_HUMAN	65.27 (50)	6.09 (6.00)	75	5.8*E* − 04	13	22

**Table 3:** Functional characterization of the 11 identified proteins found only in PCa.

SwissProt accession number	Protein name	Gene name	Subcellular location	Type of protein	Biological function
ANT3_HUMAN	Antithrombin-III OS = *Homo sapiens* GN = SERPINC1 PE = 1 SV = 1	SERPINC1	Extracellular space	Enzyme	Serine protease inhibitor in plasma that regulates the blood coagulation cascade

AMY1_HUMAN	Alpha-amylase 1 OS = *Homo sapiens* GN = AMY1A PE = 1 SV = 2	AMY1	Extracellular space	Enzyme	Carbohydrate metabolic process

UTP18_HUMAN	U3 small nucleolar RNA-associated protein 18 homolog OS = *Homo sapiens* GN = UTP18 PE = 1 SV = 3	UTP18	Nucleus	Other	It is involved in nucleolar processing of pre-18S ribosomal RNA

TYPH_HUMAN	Thymidine phosphorylase OS = *Homo sapiens* GN = TYMP PE = 1 SV = 2	TYMP	Extracellular space	Growth factor	It has a role in maintaining the integrity of the blood vessels, growth promoting activity on endothelial cells, angiogenic activity in vivo, and chemotactic activity on endothelial cells in vitro and catalyzes the reversible phosphorolysis of thymidine

ENDD1_HUMAN	Endonuclease domain-containing 1 protein OS = *Homo sapiens* GN = ENDOD1 PE = 1 SV = 2	ENDOD1	Extracellular space	Enzyme	It may act as a DNase and a Rnase

RFFL_HUMAN	E3 ubiquitin-protein ligase rififylin OS = *Homo sapiens* GN = RFFL PE = 1 SV = 1	RFFL	Cytoplasm	Enzyme	It regulates several biological processes through the ubiquitin-mediated proteasomal degradation of various target proteins and negatively regulates the tumor necrosis factor-mediated signaling pathway and p53/TP53 through its direct ubiquitination and targeting to proteasomal degradation

QORL1_HUMAN	Quinone oxidoreductase-like protein 1 OS = *Homo sapiens* GN = CRYZL1 PE = 1 SV = 2	CRYZL1	Cytoplasm	Enzyme	Quinone metabolic process

ILF2_HUMAN	Interleukin enhancer-binding factor 2 OS = *Homo sapiens* GN = ILF2 PE = 1 SV = 2	ILF2	Nucleus	Transcription regulator	It functions predominantly as a heterodimeric complex with ILF3. This complex may regulate transcription of the IL2 gene during T-cell activation

TMIG1_HUMAN	Transmembrane and immunoglobulin domain-containing protein 1 OS = *Homo sapiens* GN = TMIGD1 PE = 2 SV = 1	TMIGD1	Other	Other	Integral component of membrane

RAB36_HUMAN	Ras-related protein Rab-36 OS = *Homo sapiens* GN = RAB36 PE = 2 SV = 2	RAB36	Cytoplasm	Enzyme	Protein transport. It is probably involved in vesicular traffic

TPD52_HUMAN	Tumor protein D52 OS = *Homo sapiens* GN = TPD52 PE = 1 SV = 2	TPD52	Cytoplasm	Other	B cell differentiation and anatomical structure morphogenesis and secretion

## Abbreviations

1D: One-dimensional
2D: Two-dimensional
2D PAGE: Two-dimensional polyacrylamide gel electrophoresis
ACN: Acetonitrile
BSA: Bovine serum albumin
CHAPS: 3-[(3-Cholamidopropyl)dimethylammonio]-1-propanesulfonate
Da: Dalton
DTT: Dithiothreitol
GO: Gene Ontology
IAA: 2-Iodoacetamide
IPA: Ingenuity Pathways Analysis
LC: Liquid chromatography
MALDI-TOF-TOF: Matrix-assisted laser desorption/ionization-time of flight-time of flight
MS: Mass spectrometry
PSA: Prostate-specific antigen
SDS: Sodium dodecyl sulfate
SDS-PAGE: Sodium dodecyl sulfate polyacrylamide gel electrophoresis
TFA: Trifluoroacetic acid
Tris: Tris(hydroxymethyl)aminomethane
UniProtKB: UniProt Knowledgebase.
